# Dose–Response Relationship of Prenatal Mercury Exposure and IQ: An Integrative Analysis of Epidemiologic Data

**DOI:** 10.1289/ehp.9303

**Published:** 2007-01-11

**Authors:** Daniel A. Axelrad, David C. Bellinger, Louise M. Ryan, Tracey J. Woodruff

**Affiliations:** 1 U.S. Environmental Protection Agency, Office of Policy, Economics, and Innovation, Washington, DC, USA; 2 Department of Neurology, Harvard Medical School and Children’s Hospital Boston and; 3 Department of Environmental Health, Harvard School of Public Health, Boston, Massachusetts, USA; 4 Department of Biostatistics, Harvard School of Public Health, Boston, MA, USA; 5 U.S. Environmental Protection Agency, Office of Policy, Economics and Innovation, San Francisco, California, USA

**Keywords:** Bayesian hierarchical model, benefits, dose–response model, epidemiology, IQ, mercury, neurodevelopmental effects, noncancer risk assessment, prenatal exposure

## Abstract

**Background:**

Prenatal exposure to mercury has been associated with adverse childhood neurologic outcomes in epidemiologic studies. Dose–response information for this relationship is useful for estimating benefits of reduced mercury exposure.

**Objectives:**

We estimated a dose–response relationship between maternal mercury body burden and subsequent childhood decrements in intelligence quotient (IQ), using a Bayesian hierarchical model to integrate data from three epidemiologic studies.

**Methods:**

Inputs to the model consist of dose–response coefficients from studies conducted in the Faroe Islands, New Zealand, and the Seychelles Islands. IQ coefficients were available from previous work for the latter two studies, and a coefficient for the Faroe Islands study was estimated from three IQ subtests. Other tests of cognition/achievement were included in the hierarchical model to obtain more accurate estimates of study-to-study and end point–to–end point variability.

**Results:**

We find a central estimate of −0.18 IQ points (95% confidence interval, −0.378 to −0.009) for each parts per million increase of maternal hair mercury, similar to the estimates for both the Faroe Islands and Seychelles studies, and lower in magnitude than the estimate for the New Zealand study. Sensitivity analyses produce similar results, with the IQ coefficient central estimate ranging from −0.13 to −0.25.

**Conclusions:**

IQ is a useful end point for estimating neurodevelopmental effects, but may not fully represent cognitive deficits associated with mercury exposure, and does not represent deficits related to attention and motor skills. Nevertheless, the integrated IQ coefficient provides a more robust description of the dose–response relationship for prenatal mercury exposure and cognitive functioning than results of any single study.

Prenatal exposure to mercury through maternal consumption of fish has been associated with reduced performance on tests of neurologic function in children, including tests of cognitive development, attention and behavior, and motor skills. A comprehensive review of the mercury literature conducted by the National Research Council (NRC) Committee on the Toxicological Effects of Methylmercury concluded that, based on the evidence available, “neurodevelopmental deficits are the most sensitive, well-documented effects” of exposure to mercury (NRC 2000).

The NRC committee’s conclusion was based primarily on its review of epidemiologic studies conducted in the Faroe Islands (Grandjean et al. 1997), New Zealand (Crump et al. 1998; Kjellstrom et al. 1989), and the Seychelles Islands (Davidson et al. 1998; Myers et al. 2003). These three populations were selected for study in large part because fish consumption was known to be relatively high; human methylmercury exposure is largely attributable to intake of methyl-mercury that has accumulated in fish tissue.

All three studies measured prenatal exposure to mercury and neurodevelopmental end points in the children, though there were differences in the tests used to measure potential neurodevelopmental deficits. The Faroe Islands and New Zealand studies found a statistically significant relationship between higher prenatal mercury exposure and poorer scores on tests of neurologic function, but the Seychelles study did not. The NRC committee determined that “each of the studies was well designed and carefully conducted, and each examined prenatal MeHg [methylmercury] exposures within the range of the general U.S. population exposures” (NRC 2000).

The U.S. Environmental Protection Agency (EPA) developed a reference dose (RfD) for methylmercury that draws on the NRC analysis of data from all three epidemiologic studies. An RfD is an estimate of a daily exposure to the human population (including sensitive subgroups) that is likely to be without an appreciable risk of deleterious effects during a lifetime. However, the U.S. EPA’s review also indicates that “no evidence of a threshold arose for methylmercury-related neurotoxicity within the range of exposures in the Faroe Islands study” (U.S. EPA 2001). In addition, the RfD does not provide information about the dose–response relationship between prenatal mercury exposure and related neurologic effects, because it focuses on a single exposure level and does not identify the risk associated with that level. A dose–response model is needed to estimate the potential risk of neurodevelopmental effects in the population and the benefits of any efforts to reduce mercury exposure.

We applied a Bayesian hierarchical model to integrate the findings from the three epidemiologic studies and estimate a dose–response relationship between maternal mercury body burden and subsequent childhood decrements in intelligence quotient (IQ). We selected IQ for dose–response modeling because data related to IQ were available from all three studies, and because methods for economic valuation of IQ decrements are well established, as applied in the U.S. EPA’s previous benefits analyses for lead (U.S. EPA 1997).

## Methods

### Selection of end points and coefficients

All cognitive end points reported in the Faroe Islands (testing at 7 years of age), New Zealand (6 years of age) and Seychelles (9 years of age) studies were considered for inclusion in the hierarchical model. Neurodevelopmental tests conducted in each of the three studies at these ages are listed in the Supplemental Material (online at http://www.ehponline.org/docs/2007/9303/suppl.pdf), and those selected for our statistical model are shown in Table 1. For this analysis, we assumed a linear relationship between mercury body burdens and neurodevelopmental outcomes, in keeping with the recommendation of the NRC committee (NRC 2000). In the New Zealand and Seychelles studies, all information necessary for our model was obtained from the published papers, including linear regression coefficients (Crump et al. 1998; Myers et al. 2003). The Faroe Islands publications, however, reported results with cord blood and maternal hair mercury transformed to the log scale and provided no results of linear models (Grandjean et al. 1997, 1999). A report by the Faroe Islands investigators (Budtz-Jorgensen et al. 2005) prepared at our request provides the additional details needed for our analysis.

For the New Zealand study, two sets of dose–response coefficients were reported (Crump et al. 1998): one with the complete cohort, and the other for which one very influential observation, with unusually high maternal hair mercury, was excluded. The NRC committee reviewed the influence of the one observation and determined that exclusion of this outlier was reasonable and appropriate (NRC 2000). Our primary analysis used the coefficients from the regression in which the outlier child was excluded; coefficients for the case in which this child is included were considered in a sensitivity analysis.

For several tests and end points, results for multiple scores were reported. To avoid over-representing any particular test and to avoid adding additional complexity to our modeling, we chose only one score for a test in such cases. For example, the Faroe Islands study presents regression coefficients for the effect of mercury on four tasks of the California Verbal Learning Test, and the Seychelles study provides results for two of these. We selected the short delay recall task, which was common to both studies.

### Rescaling

Our next step was to rescale all the estimated regression coefficients and standard errors so that they correspond to test scores with the same distribution as Full-Scale IQ (that is, an SD of 15). This rescaling allows all inputs and outputs of our model to be expressed in terms of the decrement in IQ associated with a one-unit increase in mercury. Rescaling involves multiplication by a factor inversely proportional to the observed standard deviation of the score for each test. Details are provided in the Supplemental Material (online at http://www.ehponline.org/docs/2007/9303/suppl.pdf).

We also rescaled to adjust for differences in mercury biomarkers used in the studies. The New Zealand and Seychelles studies report results in terms of parts per million hair mercury, whereas results of the Faroe Islands study with the linear coefficients required for this study are reported in terms of parts per billion cord blood mercury. To combine results across studies, we converted the Faroe Islands results to their equivalents in units of hair mercury using the reported median ratio of mercury in hair to mercury in cord blood in the Faroe Islands study population, which was approximately 200 (Budtz-Jorgensen et al. 2004a).

### IQ tests in the three studies and IQ coefficient for Faroe Islands study

The Wechsler Intelligence Scales for Children (WISC) is a standard test of childhood IQ that was used in each of the three studies. The version of the test administered in the Seychelles Islands (3rd ed.; WISC-III) was different from the earlier version used in New Zealand and the Faroe Islands (revised ed.; WISC-R). In a sample of approximately 200 children, the correlation between the Full-Scale IQ scores for the two versions was 0.89; thus the WISC-R and WISC-III appear to measure the same constructs and generate scores with similar dispersion (Wechsler 1991).

The WISC-R includes 10 core subtests and three supplementary subtests. For the Faroe Islands study, the investigators administered only three subtests of the WISC-R: Digit Span and Similarities (core subtests) and Block Design (a supplementary subtest). We used data for these three subtests to estimate an IQ–mercury coefficient for the Faroe Islands cohort. This approach is supported by the findings of Sattler (1988), who identified the combinations of the 10 core subtests that provide the most valid estimates of Full-Scale IQ. Of the 45 possible combinations of two core subtests (i.e., 10 subtests taken two at a time), the combination of Similarities and Block Design ranked third in the magnitude of the validity coefficient (0.885). It is reasonable to expect that adding the information about Full-Scale IQ conveyed by the Digit Span score would produce an even higher validity coefficient. This indicates that combining the scores of the Faroese children on Similarities, Block Design, and Digit Span will provide valid estimates of their Full-Scale IQ scores.

Regression coefficients and standardized coefficients (coefficient as percent of corresponding response standard deviation) for the three subtests are shown in Table 2. At our request, the Faroe Islands investigators fit data for these three subtests in a structural equation model (SEM) to estimate a standardized coefficient for a hypothetical Full-Scale IQ (Budtz-Jorgensen et al. 2005). Structural equation modeling allows the combination of multiple exposures and responses via the use of latent variables (Budtz-Jorgensen et al. 2002). In the SEM analysis of IQ, the three WISC-R subtests are viewed as representative of an underlying latent IQ variable.

When fitting an SEM, it is necessary to specify the scaling of any latent variables involved in the model. The Faroe Islands investigators assumed that the IQ latent variable was on the same scale as Digit Span. The analysis estimated a coefficient of −0.024 and a standard error of 0.011 for the effect of each 10 ppb of cord blood mercury on latent Full-Scale IQ, with a *p*-value of 0.031 (Budtz-Jorgensen et al. 2005).

As with the general case discussed above, the coefficient of the SEM latent variable also requires rescaling so that it is comparable to Full-Scale IQ. In this particular case, there are two possible approaches to rescaling (Table 2): One uses the standard deviation for Digit Span (because the latent variable is assumed to be on the same scale as Digit Span), whereas the second uses the estimated standard deviation of the latent variable itself, obtained as part of the SEM fitting procedure. Our primary approach uses the IQ estimate derived with the Digit Span standard deviation. However, the estimate derived with the standard deviation of the SEM latent variable may also be valid, so we used this estimate in a sensitivity analysis. The standard deviation for the SEM latent variable is considerably smaller than the Digit Span standard deviation, resulting in a larger estimated impact of mercury exposure on IQ for the Faroe Islands cohort.

### Statistical modeling

To estimate the association between mercury and IQ using information from the three studies, we used a hierarchical random-effects model that includes study-to-study as well as end point–to–end point variability. Such models are commonly used in settings where the goal is to combine related information from several different sources. For example, Dominici et al. (2000) used such a model to combine dose–response data related to particulate matter from different U.S. cities. The approach used here extends the Dominici work by including random effects that reflect two levels of variability. Our model is similar to the one described by Coull et al. (2000) in their response to Dominici et al. [see also Coull et al. (2003)].

Our analysis can be described as follows: Let *b*_1_, *b*_2_,….*b*_L_ represent the set of L estimated standardized regression coefficients that we wish to analyze in a combined model. Similarly, we index the associated standard errors as *s*_1_, *s*_2_,….*s*_L_. Along with each *b*_i_ we assign a covariate *study_i_*, which takes the value 1, 2 or 3 and indicates whether the coefficient came from New Zealand, Seychelles, or the Faroe Islands study, respectively. We also assign another covariate *endpoint*_i_ that indicates which particular developmental end point the coefficient *b*_i_ was based on. We then fit the model


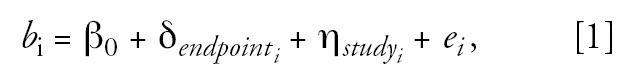


where β_0_ is the overall mean, *e*_i_ is a random error term assumed to be normally distributed with mean 0 and known variance *s_i_*^2^, η*_studyi_* is a study-specific random effect, assumed to be normally distributed with mean 0 and variance σ*_study_*^2^, and δ*_endpointi_* is an end point–specific normal random effect with mean 0 and variance σ*_endpoint_*^2^.

Although it is technically feasible to fit our model using maximum-likelihood estimation, the limited data meant that there was little information available to reliably estimate the variance components. Instead we implement the model with a Bayesian approach. Maximum-likelihood estimation is based on so-called frequentist inference, which refers to the properties of estimators and random variables under hypothetical replications of the experiment that generated the data. For example, a sample mean will hover around the true but unknown population mean under repeated sampling from the population. Frequentist inference treats model parameters as fixed, albeit unknown, quantities to be estimated. In contrast, a Bayesian approach treats not only the data but also all unknown model parameters as random variables. Thus, Bayesian inference requires specification not only of the probability distribution of the data, but also the probability distributions (priors) of model parameters.

In recent years, advances in computational tools for Bayesian modeling have led to vastly increased usage of these methods. The most widespread computational approach, Markov Chain Monte Carlo, has been implemented in the user-friendly package WinBUGS (Lunn et al. 2000). WinBUGS has become popular even among frequentist statisticians because, when sample sizes are large and the assumed distributions on unknown model parameters are very broad (i.e., noninformative or “flat” priors), Bayesian inference will provide results very close to those obtained through a frequentist approach (Carlin and Louis 2000). WinBUGS is particularly useful for fitting complex hierarchical models that would be difficult to handle using a maximum-likelihood approach, which was important to our decision to fit our models with a Bayesian approach. Additionally, as discussed below, the approach allowed us to overcome some computational problems through the use of slightly informative priors. Our analysis used WinBUGS version 1.4 (http://www.mrc-bsu.cam.ac.uk/bugs/).

The Bayesian approach requires the specification of prior distributions for all model parameters, including β_0_, σ*_study_*^2^, and σ*_endpoint_*^2^ Data limitations in our setting precluded the specification of fully noninformative priors on the variance components. To address this concern, we reparameterized to


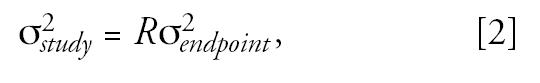


so that *R* is a ratio of study-to-study variability relative to end point–to–end point variability. We then fitted the model for various fixed, reasonable values of *R*.

Although it is common for Bayesian modelers to use an inverse gamma to specify a prior distribution on a variance component, we found this formulation to be unstable in that our results were highly sensitive to the gamma parameters. This finding is consistent with a number of reports in the literature (e.g., Gelfand et al. 1995). Gelman (2006) argues that more stable results can be obtained by specifying priors directly on the variance components or their square root. We used this approach, with appropriate prior distributions determined by examination of a profile likelihood surface obtained by treating the parameters *R* and σ*_study_* as fixed and known. This analysis found that although there was little information in the data to estimate *R*, the most likely values for σ*_study_* ranged between 0 and 0.2. We therefore specified a uniform prior on σ*_study_* with this range.

All fitted models were checked for convergence and refit with different starting values to ensure that reliable estimates had been obtained. These procedures yielded computationally stable results and allowed us to explicitly evaluate the sensitivity of our results to the values of the variance components. Sample code is provided in the Supplemental Material (online at http://www.ehponline.org/docs/2007/9303/suppl.pdf).

In the frequentist approach to statistical analysis, confidence intervals (CIs) are typically based on a normality assumption and, in the case of a 95% confidence interval, correspond to the estimated parameter ± 1.96 times the standard error. A confidence interval is based on the probability distribution of the estimated parameter, and should not be interpreted as a probability statement about the parameter of interest, which is assumed to be fixed (nonrandom) but unknown. In contrast, because a Bayesian approach treats model parameters as random variables, the distribution of the unknown parameter of interest can be computed. This distribution is known as the posterior, and the highest posterior density (HPD) interval refers to the most probable range of the parameter of interest, given the observed data. In settings where sample sizes are large and flat priors have been used, confidence and HPD intervals will generally be indistinguishable. Although our Bayesian analysis yields HPD intervals, we refer to these as confidence intervals to aid in the interpretation of our results.

Further discussion of our modeling process may be found in a separate paper (Ryan LM, in press).

### Sensitivity analyses

We conducted several sensitivity analyses to examine the impacts of alternate input data. The first sensitivity analysis considers a model that includes only the IQ dose–response coefficients estimated for the three studies. Maximum-likelihood estimation was straightforward in this case, because no end point–to–end point variation was involved.

Other sensitivity analyses used the Bayesian approach to incorporate alternate input values. We considered the use of coefficients from the New Zealand study in which a single highly exposed child is included. We also repeated the analysis using the alternate estimate of the rescaled IQ dose–response coefficient for the Faroe Islands study, where the rescaled coefficient uses the standard deviation of the latent variable from the SEM.

## Results

### Primary analysis

Table 3 shows the cognitive end points from each of the three studies used in this analysis, the regression coefficients reported in the three studies, and coefficients rescaled so that they are all expressed in comparable terms (i.e., rescaled using the standard deviation of IQ, and with exposure expressed in terms of hair mercury).

**Figure 1:**
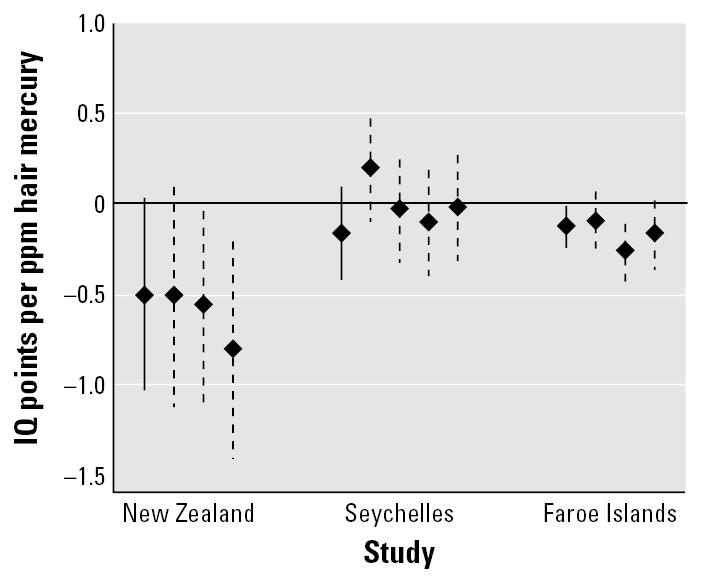
Coefficients and 95% confidence intervals for the dose–response relationship between neurodevelopmental test scores and maternal hair mercury from three epidemiologic studies. Solid lines indicate coefficients for Full-Scale IQ, and dashed lines indicate coefficients for other neurodevelopmental tests included in the primary analysis (see Table 3). Coefficients for end points other than IQ are rescaled to be expressed in equivalent terms.

Using values of *R* (ratio of study-to-study variability relative to end point–to–end point variability) between 0.25 and 4 produced central estimate dose–response coefficients ranging from −0.15 (*R* = 0.25) to −0.19 (*R* = 4.0) IQ points per parts per million of maternal hair mercury, which were statistically significant in all cases (Table 4). As *R* increases, the study-to-study variance component also increases (Table 4). Although there is not enough information available to reliably estimate both *R* and σ*_study_*, visual inspection of the data displayed in Figure 1 suggests that there is likely to be more study-to-study than end point–to–end point variation. Because the results appear to stabilize at a value of *R* = 3.0 and because this value seems reasonable, we use this value for all subsequent analysis and as the basis of our main study findings.

**Figure 2:**
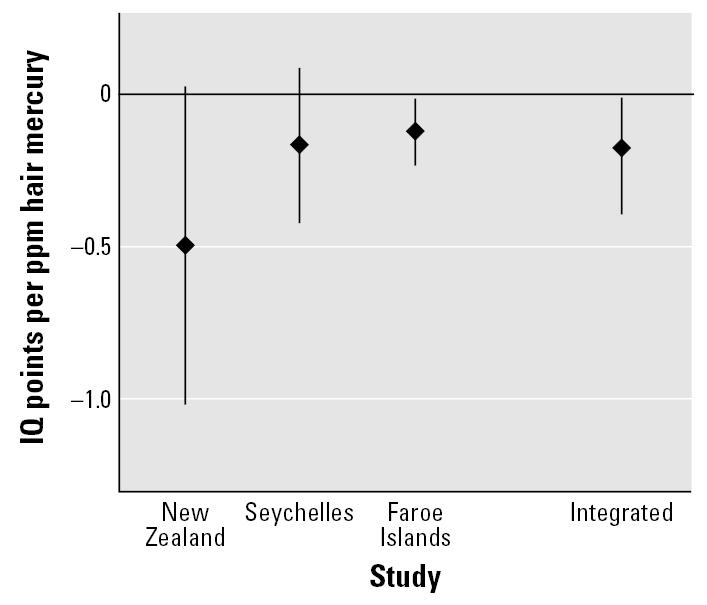
Coefficients and 95% confidence intervals for the dose–response relationship between IQ and maternal hair mercury from the three epidemiologic studies and for the results of the integrated analysis.

The integrated analysis produced a central estimate of −0.18 (95% CI, −0.378 to −0.009) IQ points for each part per million maternal hair mercury, similar to the results found for both the Faroe Islands and Seychelles studies, and lower than the estimate found in the New Zealand study (Figure 2).

### Sensitivity analyses

Our first sensitivity analysis, using simple maximum-likelihood analysis, includes only the IQ dose–response coefficients from the three studies, and does not include the other cognitive outcomes. We find an overall mean dose–response coefficient of −0.145 (95% CI, −0.259 to −0.047) (Table 5). Note that the study-to-study variance component had an estimated value of 0 in this analysis.

Using the New Zealand coefficients with the outlier included in the hierarchical analysis reduces the central estimate of the dose–response coefficient, compared with the primary analysis, to −0.125 (Table 5). The study-to-study variance component was reduced, and the precision associated with the coefficient increased, with 95% CI of −0.236 to −0.007).

The Bayesian model was also rerun with the alternate estimate of the IQ dose–response coefficient for the Faroe Islands, where the IQ coefficient from the SEM analysis was rescaled using the estimated standard deviation of the latent variable (0.586) rather than the standard deviation of the Digit Span subtest (1.45). This produced a rescaled Faroe Islands dose–response coefficient of −0.307, compared with the rescaled coefficient of −0.124 used as input to the primary analysis. The resulting integrated dose–response coefficient for IQ increases in magnitude to −0.25 (95% CI, −0.491 to −0.052) (Table 5).

## Discussion

Our analysis integrated data from three epidemiologic studies to estimate a change in childhood IQ of −0.18 IQ points (95% CI, −0.378 to −0.009) for every part per million mercury in maternal hair. This central estimate is relatively close to the values for the Faroe Islands and Seychelles studies, suggesting less influence on the integrated value from the larger coefficient estimated in the New Zealand study. The smaller influence of the New Zealand coefficient is attributed to the smaller size of the cohort and the greater uncertainty in the central estimate of the dose–response coefficient, as depicted in Figure 2.

Our analysis provides the ability to estimate benefits from reductions in mercury exposure, similar to previous analyses estimating benefits of reducing childhood lead exposure. We assume a linear, nonthreshold relationship between prenatal mercury exposure and IQ deficits in the children. The choice of a linear nonthreshold dose–response model was based on several considerations: the shape of the dose–response in the range of the observed data; the magnitude of the extrapolation below the observed data; relevant biologic considerations; and the available information for the Seychelles and New Zealand studies, which consisted of linear dose–response coefficients. The NRC panel concluded that linear models are most appropriate for dose–response modeling of mercury’s neurodevelopmental effects in the absence of persuasive evidence supporting an alternative functional form (NRC 2000). In addition, the U.S. EPA has concluded that “no evidence of a threshold arose for methylmercury-related neurotoxicity within the range of exposures in the Faroe Islands study” (U.S. EPA 2001).

An important consideration in extrapolating below the observed data is the extent of the extrapolation. The lowest exposure in the Faroe Islands study is 0.9 ppb mercury in cord blood, equivalent to 0.53 ppb mercury in maternal blood [assuming a ratio of mercury in cord blood to maternal blood equal to 1.7 (Stern and Smith 2003)]. More than 50% of U.S. women had blood mercury concentrations > 0.53 ppb in 1999–2002 (Centers for Disease Control and Prevention 2004). Although there is limited information on the shape of the dose–response relationship at lower exposure levels, it is reasonable to assume that the linear dose–response relationship recommended by the NRC for the observed range of the data in the epidemiologic studies applies as well in extrapolating to the range of the U.S. data. Scientific findings suggest that the slope of the dose–response curve may in fact be steeper at lower doses (i.e., supralinear). A log-linear model was found to provide the best fit between cord blood mercury and cognitive effects in the Faroe Islands study (Budtz-Jorgensen et al. 2000). Also, analyses of the relationship between childhood lead exposure and IQ have found a steeper response at exposures < 10 μg/dL (Lanphear et al. 2005), and other findings in the literature suggest the plausibility of supralinear dose–response relationships (Castorina and Woodruff 2003; Thompson and Myers 2006). If such a relationship applies in the case of mercury and IQ, a linear term will underestimate the effect.

In addition, recent commentaries have proposed using linear dose–response models for noncancer end points (Clewell and Crump 2005; Crawford and Wilson 1996). The authors note that assuming linearity is a reasonable approach, based on similar considerations underpinning linear nonthreshold dose–response models for carcinogens, including the presence of background biologic processes, background exposures to other chemicals, and variability in human response (Clewell and Crump 2005; Crawford and Wilson 1996; Crump et al. 1976). Considering all of the available information, the assumption of linearity in our analysis is a reasonable approach.

Full-Scale IQ is a composite index that averages a child’s performance across many functional domains, providing an overall picture of cognitive health. IQ as measured at school age has been shown to be predictive of later outcomes such as academic and occupational success (Neisser et al. 1996). However, if mercury affects only specific cognitive functions, using Full-Scale IQ as the end point for a benefits analysis will underestimate the neurodevelopmental impacts on other targeted functions.

Moreover, there may be substantial deficits in cognitive well-being even in individuals with normal or above average IQ. For example, two of the most sensitive end points in the Faroe Islands study were the Boston Naming Test (BNT), which assesses word retrieval, and the California Verbal Learning Test (CVLT), which assesses the acquisition and retention of information presented verbally. A child who has deficits in either of these skills could, depending on their severity, be at a considerable disadvantage in the classroom and at substantial educational risk. Neither of these abilities is directly assessed by the WISC IQ test, however, so they do not explicitly contribute to a child’s IQ score. Therefore, benefits calculations relying solely on IQ decrements are likely to underestimate the benefits to cognitive functioning of reduced mercury exposures. In addition, impacts on other neurologic domains (such as motor skills and attention/behavior) are not represented by IQ scores and thus are also excluded from the analysis.

An earlier version of this work, included in the technical documentation for the U.S. EPA’s Clean Air Mercury Rule of March 2005, reported a central estimate of −0.13 for the mercury–IQ coefficient (Bellinger 2005; Ryan 2005; U.S. EPA 2005). A revised version, reflecting corrections to the original model code, was included in subsequent rule-making documentation, and reports a central estimate of −0.16 (U.S. EPA 2006). The central estimate of −0.18 reported here reflects revisions that adapt the recommendations of Gelman (2006) for specification of prior assumptions in Bayesian analysis, as discussed above.

Although exploratory likelihood-based analysis indicated a likely range for the prior distribution on σ*_study_* of 0–0.2, sensitivity of the model to the uniform prior distribution on σ*_study_* was assessed by changing the specification to a range of 0–0.3 or 0–0.4. Posterior estimates of σ*_study_* increased slightly. The estimated dose–response coefficient remained stable at approximately −0.18, and there was some variation in width of the confidence intervals, with upper confidence limits marginally exceeding zero with the broader priors.

This analysis relies on use of summary statistics for each of the three studies. Original data were not available for this analysis. Although a lack of original data is a potential limitation, its impact here is lessened for several reasons. All three epidemiologic studies had careful prospective designs and measured a variety of important potential confounders. The dose–response coefficients were derived from well-documented regression models that adjusted for age, maternal education, and other important factors. Dominici et al. (2000) took a similar approach for hierarchical modeling of estimated dose–response coefficients extracted from separate studies.

We converted the Faroe Islands cord blood mercury coefficients to hair mercury units using the study’s median hair:cord blood ratio of 200. However, this ratio is not constant over the range of exposures (Budtz-Jorgensen et al. 2004a). To evaluate the impact of the varying ratio, we conducted a simulation using parameters reported by Budtz-Jorgensen et al. (2004a). The simulation produced multiple paired estimates, each consisting of a direct hair mercury–IQ coefficient and an indirect hair mercury–IQ coefficient derived by estimating a cord blood mercury–IQ coefficient then dividing by the hair:cord blood ratio of 200. On average, the direct estimate of the hair mercury–IQ coefficient was around 10% smaller than the indirect estimate. Using a constant ratio therefore had a small impact on the estimated hair mercury–IQ coefficient for the Faroe Islands study; the impact on the integrated coefficient derived from all three studies would be even smaller.

We focused the selection of outcomes for this analysis on tests of cognitive functioning. We did evaluate an alternative formulation of the model that included tests of attention, behavior, and motor skills (Ryan 2005). Not surprisingly, the results of this model displayed greater uncertainty than the primary analysis, indicating that the overall signal is dampened if we include end points unrelated to cognition.

An advantage of our hierarchical modeling approach is that it can produce separate dose–response coefficients for each of the outcomes included in the model, as well as a coefficient integrating all outcomes. An important reason for focusing on IQ was that methods already exist for valuing this end point in economic benefit–cost analysis. However, it would be useful and appropriate for economic analyses to consider a broader range of outcomes. For example, in the primary analysis model, the overall mean coefficient for the achievement/cognition domain is −0.19 (95% CI, −0.394 to −0.021), and the coefficient for the BNT is −0.21 (95% CI, −0.443 to −0.037). These additional dose–response estimates should be considered for use in expanded economic analyses of neurodevelopmental effects; this would require economic research on individuals’ willingness-to-pay for reducing risks of neurodevelopmental effects.

A recent article by Trasande et al. (2005) used results from Budtz-Jorgensen et al. (2005) to estimate a Faroe Islands IQ decrement of 4–8% of a standard deviation for each 10 ppb cord blood mercury. The comparable Faroe Islands estimate in our analysis is either 1.65 or 4.10% of a standard deviation (Table 2). The Trasande estimate is based on the results for the BNT, the CVLT (both tests of cognitive function), and the Continuous Performance Test (test of attention/behavior, and which provided the upper end of the range). Trasande et al. (2005) did not consider the results of the WISC subtests (for which the decrements generally equal around 2% of a standard deviation), which are most directly relevant to estimation of the mercury–IQ relationship. Our estimate is based on the SEM result that was derived from the three WISC subtests.

Finally, the integrated dose–response analysis assumes that the exposures assigned to each study subject are accurate representations of true exposure. In reality, there is likely to be some discrepancy between measured and actual exposures—for example, due to variation in hair length. Alternatively, the true exposure of interest may have occurred during the first trimester of pregnancy, whereas mercury in maternal hair samples only a few centimeters in length collected at birth and in cord blood samples reflect exposures later in pregnancy. Presence of exposure measurement error could introduce a bias in the results, most likely toward the null (Budtz-Jorgensen et al. 2004b).

Using a statistical technique that accounts for variability within and between studies, we have produced an integrated estimate of the dose–response relationship between prenatal mercury exposure and IQ. IQ does not represent all neurodevelopmental deficits associated with mercury, so estimates of effects using this relationship will understate the overall impacts of prenatal mercury exposure. Nevertheless, the estimated mercury–IQ relationship provides a broad-based measure of effects on cognitive development and can be readily applied to estimate benefits of reducing mercury exposures in the population.

## Correction

In the Abstract, the sections “Primary analysis” and “Discussion,” and Table 5, the 95% CI for estimate of childhood IQ was −0.387 to −0.012 in the original manuscript published online. It has been corrected here.

## Figures and Tables

**Table 1:** Cognitive tests included in the primary mercury–IQ dose–response analysis model.

Test	Domain assessed
Faroe Islands study (917 children tested at 7 years of age)	
WISC-R (Full-Scale IQ^a^)	General intelligence
CVLT (short term)	Verbal learning and memory
Bender-Gestalt Test (errors on copying)	Visual–motor integration
BNT (no cues)	Confrontational naming
New Zealand study (237 children tested at 6 years of age)	
WISC-R (Full-Scale IQ)	General intelligence
WISC-R (Performance IQ)	General intelligence
MCC (Perceptual)	General development
TOLD (spoken language quotient)	General verbal skills
Seychelles study (643 children tested at 9 years of age)	
WISC-III (Full-Scale IQ)	General intelligence
CVLT (short term)	Verbal learning and memory
VMI	Visual–motor integration
BNT (total score)	Confrontational naming
WRAML (design memory)	Visual memory

Abbreviations: BNT, Boston Naming Test; CVLT, California Verbal Learning Test; MCC, McCarthy Scales of Children’s Abilities; TOLD, Test of Language Development; VMI, Developmental Test of Visual–Motor Integration; WISC-R, Wechsler Intelligence Scales for Children, Revised; WISC-III, Wechsler Intelligence Scales for Children, 3rd ed.; WRAML, Wide Range Assessment of Memory and Learning.

a Effect of prenatal mercury exposure on Full-Scale IQ, as derived from a structural equation model of the three WISC-R subtests conducted in the Faroe Islands study (Digit Span, Similarities, Block Design).

**Table 2:** Estimated regression coefficients for the three WISC-R subtests conducted in the Faroe Islands study, and SEM-derived estimates of Full-Scale IQ.

End point	β (SE)^a^	Response SD^b^	Standardized coefficient^c^
WISC-R subtests			
Digit Span	−0.025 (0.018)	1.45	−1.72
Similarities	−0.039 (0.050)	3.86	−1.01
Block Design	−0.175 (0.098)	8.92	−1.94
SEM estimates of WISC-R Full-Scale IQ			
Estimate A	−0.024 (0.011)	1.45	−1.65
Estimate B	−0.024 (0.011)	0.586	−4.10

Abbreviations: SEM, structural equation model.

a Effect per 10-ppb increase in cord blood mercury, as reported by Budtz-Jorgensen et al. (2005).

b The response SD for the three WISC-R subtests is the SD for the Faroe Islands cohort for each subtest. For the SEM estimates, two choices are available: Estimate A assumes that the response SD for Digit Span applies to the SEM estimate, because the SEM latent variable representing IQ was estimated under the assumption that it has the same scale as the Digit Span end point. Estimate B uses the SD of the SEM latent variable itself, obtained as part of the SEM estimation procedure. Estimate A is used in the primary analysis, and Estimate B is applied in a sensitivity analysis.

c The standardized coefficient is the estimated coefficient (β) as a percentage of the response SD.

**Table 3:** Original and rescaled regression coefficients and associated standard errors for cognitive end points from the Faroe Islands, New Zealand, and Seychelles studies of prenatal mercury exposure.

			Original scale		Rescaled^a^	
Study	End point	Scaling factor^a^	β	SE	β	SE
Primary analysis inputs						
Faroe Islands (Budtz-Jorgensen et al. 2005)	Full-Scale IQ^b^	5.17	−0.024	0.011	−0.124	0.057
	Bender^c^	−1.42	0.073	0.059	−0.104	0.083
	BNT	1.37	−0.190	0.063	−0.260	0.086
	CVLT	2.91	−0.058	0.032	−0.169	0.093
New Zealand, outlier excluded^d^ (Crump et al. 1998)	Full-Scale IQ	0.94	−0.53	0.285	−0.50	0.268
	Performance IQ	0.94	−0.54	0.330	−0.51	0.310
	TOLD	0.94	−0.60	0.300	−0.56	0.282
	MCC	1.5	−0.53	0.210	−0.80	0.315
Seychelles (Myers et al. 2003)	Full-Scale IQ	1.29	−0.13	0.10	−0.17	0.130
	CVLT	14.42	0.013	0.010	0.19	0.144
	BNT	3.13	−0.012	0.046	−0.038	0.144
	WRAML	5.17	−0.021	0.029	−0.109	0.150
	VMI	1.28	−0.010	0.12	−0.013	0.150
Sensitivity analysis inputs						
Faroe Islands, alternate IQ (Budtz-Jorgensen et al. 2005)	Full-Scale IQ	12.8	−0.024	0.011	−0.307	0.141
New Zealand, outlier included^d^ (Crump et al. 1998)	Full-Scale IQ	0.94	−0.18	0.155	−0.17	0.15
	Performance IQ	0.94	−0.12	0.165	−0.11	0.16
	TOLD	0.94	−0.19	0.145	−0.18	0.14
	MCC	1.5	−0.18	0.110	−0.27	0.17

Abbreviations: BNT, Boston Naming Test; CVLT, California Verbal Learning Test; MCC, McCarthy Scales of Children’s Abilities; TOLD, Test of Language Development; VMI, Developmental Test of Visual–Motor Integration; WRAML, Wide Range Assessment of Memory and Learning.

a See Supplemental Material (http://www.ehponline.org/docs/2007/9303/suppl.pdf) for derivation of scaling factors. Rescaled coefficients are interpretable in the same scale as Full-Scale IQ. For the Faroe Islands study, rescaling also converts the values from units of cord blood mercury to units of hair mercury, to be comparable with the New Zealand and Seychelles exposure metrics.

b Full-Scale IQ for the Faroe Islands is estimated with an SEM combining three WISC-R subtests (Digit Span, Similarities, Block Design). The primary estimate is scaled using the response SD of Digit Span (Table 2, Estimate A); the alternate estimate is scaled using the standard deviation of the SEM latent variable itself, obtained as part of the SEM estimation procedure (Table 2, Estimate B).

c The scaling factor for Bender is negative because higher scores on this test represent poorer performance.

d The primary analysis inputs for the New Zealand study are derived with one highly exposed child excluded; the sensitivity analysis inputs are derived with that child included. SEs for the New Zealand study are obtained by subtracting the reported regression coefficient from the reported upper confidence limit and dividing by two.

**Table 4:** Estimates of an IQ–mercury dose–response coefficient from a hierarchical model, integrating data from three epidemiologic studies, for different values of *R* = σ*_study_*^2^/σ*_endpoint_*^2^.

*R*	σ*_study_* (SE)	β_IQ_(SE)^a^	95% CI^b^
4.0	0.118 (0.051)	−0.188 (0.096)	−0.398 to −0.010
3.5	0.116 (0.050)	−0.182 (0.091)	−0.390 to −0.007
3.0	0.112 (0.051)	−0.180 (0.092)	−0.378 to −0.009
2.5	0.110 (0.051)	−0.183 (0.090)	−0.384 to −0.017
2.0	0.107 (0.050)	−0.178 (0.088)	−0.371 to −0.012
1.5	0.095 (0.053)	−0.168 (0.086)	−0.360 to −0.003
1.0	0.086 (0.051)	−0.165 (0.080)	−0.338 to −0.015
0.5	0.068 (0.046)	−0.160 (0.071)	−0.321 to −0.026
0.25	0.049 (0.038)	−0.151 (0.061)	−0.283 to −0.033

a Estimated IQ decrement per part per million maternal hair mercury. The square root of the study-to-study variance component, σ*_study_*, is assumed to have a uniform distribution with a range of 0–0.2.

b See “Statistical modeling” for discussion of how confidence intervals relate to highest posterior density intervals.

**Table 5:** Sensitivity analysis for estimation of integrated IQ dose–response coefficients.

Analysis	σ*_study_*(SE)	β_IQ_(SE)	95% CI^a^
Primary analysis	0.112 (0.051)	−0.180 (0.092)	−0.378 to −0.009
Use only IQ coefficients^b^	0.0 (NA^c^)	−0.145 (0.051)	−0.259 to −0.047
Include New Zealand outlier^d^	0.056 (0.042)	−0.125 (0.056)	−0.236 to −0.007
Alternate Faroe Islands IQ^e^	0.132 (0.044)	−0.254 (0.112)	−0.491 to −0.052

NA, not applicable. The value of *R* is set to 3.0.

a See “Statistical modeling” for discussion of how confidence intervals relate to highest posterior density intervals.

b Maximum-likelihood estimation of integrated IQ dose–response coefficient considering only the IQ dose–response coefficients from the three epidemiologic studies.

c SE could not be estimated because the estimated value of σ*_study_* was on the boundary of the parameter space.

d Estimation of integrated IQ dose–response coefficient with a Bayesian hierarchical model that incorporates all end points listed in Table 1, and using alternate values for New Zealand study shown in Table 3.

e Estimation of integrated IQ dose–response coefficient with a Bayesian hierarchical model that incorporates all end points listed in Table 1, and using the alternate value for Full-Scale IQ for the Faroe Islands study shown in Table 3.
